# Bias in the estimated reporting fraction due to vaccination in the time-series SIR model

**DOI:** 10.1371/journal.pone.0330568

**Published:** 2025-08-22

**Authors:** Tiffany Leung, Matthew Ferrari

**Affiliations:** Center for Infectious Disease Dynamics, Pennsylvania State University, University Park, Pennsylvania, United States of America; Virginia Commonwealth University, UNITED STATES OF AMERICA

## Abstract

The time-series Susceptible-Infectious-Recovered (TSIR) model has been a standard tool for studying the non-linear dynamics of acute, immunizing infectious diseases. The standard assumption of the TSIR model, that vaccination is equivalent to a reduction in the recruitment of susceptible individuals, or the birth rate, can lead to a bias in the estimate of the reporting fraction and of the total incidence. We show that this bias increases with the level of vaccination due to a double counting of individuals who are infected prior to the age of vaccination. We present a simple correction for this bias by discounting the observed number of cases by the product of the number that occur prior to the average age of vaccination and the vaccination coverage during the initial susceptible reconstruction step of the TSIR model fitting. We generate a time series of measles cases using an age-structured SIR transmission model with vaccination after birth (at 9 months of age) and illustrate the bias with the standard TSIR fitting method. We then illustrate that our proposed correction eliminates the bias in the estimated reporting fraction and total incidence. We note further that this bias does not impact the estimates of the seasonality of transmission.

## Introduction

The incidence of acute, immunizing infectious diseases displays complex non-linear dynamics that have been remarkably well described by simple dynamical models [1–3]. The time-series Susceptible-Infectious-Recovered (TSIR) model [2] has been an essential tool in bridging non-linear epidemic models and empirical data on disease incidence, particularly of immunizing infectious diseases during the pre-vaccination era [2, 4–6]. The TSIR model is a discrete time version of the continuous compartmental Susceptible-Infectious-Recovered (SIR) type epidemic models based on mass-action transmission [2].

The TSIR model makes an implicit assumption that the sum of (unimmunized) births and cases will be approximately equal over time. That is, each individual born will become infected (immunized) at some point over their lifetime and fast-scale deviations in the balance of cases and births reflect the non-linear dynamics of transmission and immunity, and seasonal variation [2]. For childhood infectious diseases, such as measles, mumps, and rubella, this is likely to be a fairly weak assumption and is supported by high adolescent seroprevalence in the pre-vaccine era [7]. A powerful property of the TSIR model, that derives from this assumption, is that it does not require incidence to be perfectly observed. The under-reporting fraction is encoded in the ratio of cumulative cases to cumulative unimmunized births [2, 4, 6].

Routine infant vaccination has been incorporated into the TSIR model by modulating the birth rate of unimmunized (susceptible) individuals. Births at each time step are discounted by the fraction immunized through vaccination after accounting for effectiveness [8–11]. However, where vaccination takes place after birth, such as at 9 months of age for measles as recommended by the World Health Organization for countries where measles is common [12], a scenario arises in which an individual destined to be vaccinated may become infected in the interval following loss of maternal immunity and before vaccination age. Such an individual would be doubly counted in the TSIR model as both an immunized birth and a case, leading to the violation of the assumption that the sum of unimmunized births and cases equate each other over time. In countries where measles is uncommon, the recommended age for measles vaccination is later at 12 to 15 months [13], leaving a longer time gap still for the potential for double counting.

In this study we relax the assumption of vaccination at birth and show that this double counting in the TSIR model can lead to a bias in the estimation of reporting fractions and of disease burden in the presence of vaccination. We show that the magnitude of this bias increases with the coverage of routine vaccination and is stronger with higher birth rates. We also show that the bias does not impact the estimation of seasonality in transmission. We propose a method to correct for this bias and evaluate its performance.

## Methods

### Dynamical model of measles

We generate daily time series of measles incidence using an age-structured stochastic compartmental model of measles transmission and vaccination, with a population stratified into 40 discrete age classes (24 monthly classes up to 2 years old; 1 class from 2 to 5 years old; 14 5-year classes from 5 to 75 years old; and 1 class of 75 years and older). Individuals are either temporarily immune with maternal antibodies (M), susceptible (S), infectious (I), or recovered (R). Additionally, they are either unvaccinated (*M*_*i*_, *S*_*i*_, *I*_*i*_, *R*_*i*_) where *i* is the age class, or vaccinated ($M_{v,i}$, $S_{v,i}$, $I_{v,i}$, $R_{v,i}$) denoted by *v*. Note that being vaccinated does not necessarily mean they are immunized; that is, vaccine effectiveness may be less than 1.

Individuals are born either susceptible (*S*_0_) or temporarily immune with maternal antibodies (*M*_0_). The fraction, *q*(*t*), of the birth cohort at time *t* that is born with maternal antibodies is determined by the proportion of individuals between 10 to 55 years old that is immune, either through prior infection or vaccination [14]. We assume a constant death rate *μ* for all age classes and set it equal to the birth rate *b* for a constant population size. We used exponential rates of aging every 15 days. We assume an average infectious period (1/γ) of 2 weeks [2, 4], an average maternal immunity period (1/σ) of 4 months [15], and lifelong immunity following infection or effective vaccination.

We use an age-dependent rate of routine vaccination, shaped as a truncated normal distribution with mean of 9 months and standard deviation of 0.5 months, constrained to the interval of 0 and 24 months. The height of the normal distribution is scaled by a constant factor such that the cumulative probability of vaccination by 24 months of age is equal to the routine vaccination coverage for a given simulation. We do not model a second dose of vaccination. Individuals who are maternally immune, susceptible, or recovered may become vaccinated. We assume 100% vaccine effectiveness on susceptible individuals and no effect on maternally immune or recovered individuals. Vaccination of maternally immune individuals results in imperfect vaccine effectiveness at the population level. Vaccinating an individual who is maternally immune or recovered only changes their vaccination status. For example, upon vaccination, a susceptible individual *S*_*i*_ becomes $R_{v,i}$, and a maternally immune individual *M*_*i*_ becomes $M_{v,i}$.

For simplicity, we present the model with the following set of ordinary differential equations:


$$\frac{dM_{i}}{dt}=qb_{i}N-\sigma_{i}M_{i}-\mu_{i}M_{i},$$

$$\frac{dS_{i}}{dt}=(1-q)b_{i}N+\sigma_{i}M_{i}-\lambda_{i}S_{i}-\mu_{i}S_{i},$$

$$\frac{dI_{i}}{dt}=\lambda_{i}S_{i}-\gamma I_{i}-\mu_{i}I_{i},$$

$$\frac{dR_{i}}{dt}=\gamma I_{i}-\mu_{i}R_{i},$$

$$\frac{dM_{v,i}}{dt}=-\sigma_{i}M_{v,i}-\mu_{i}M_{v,i},$$

$$\frac{dS_{v,i}}{dt}=\sigma_{i}M_{v,i}-\lambda_{i}S_{v,i}-\mu_{i}S_{v,i},$$

$$\frac{dI_{v,i}}{dt}=\lambda_{i}S_{v,i}-\gamma I_{v,i}-\mu_{i}I_{v,i},$$

$$\frac{dR_{v,i}}{dt}=\gamma I_{v,i}-\mu_{i}R_{v,i}.$$

The force of infection for age class *i* is


$$\lambda_{i}(t)=\sum_{j=1}^{40}(1+\beta_{0}\cos(2\pi t/365+\phi ))\beta c_{i,j}(I_{j}+I_{v,j})$$

where *β* is the transmission coefficient; *c*_*i*,*j*_ is the contact rate from age group *j* to age group *i*; $\beta_{0}$ is the relative amplitude of seasonal forcing; and *ϕ* is the phase shift. For the stochastic model and the full transition matrix, see the Supplementary Information (S1 Text).

The transmission coefficient is calculated with the next-generation matrix using a basic reproduction number, *R*_0_, of 15 [16, 17] and an infectious period of 14 days [2, 4]. Case importations occur with a probability 0.003 at all time steps to maintain endemicity. We assume either homogeneous contact mixing or assortative mixing following the pattern for Kenya [18].

We simulated the system in daily time steps using the tau-leaping algorithm [19], starting with 5 infected individuals in a fully susceptible population of 1 million with an age distribution consistent with Kenya [20]. The number of events is simulated as a Poisson draw with mean *k*_*i*_, where *k*_*i*_ is the rate of event *i* per time step. If the Poisson draw is higher than what is feasible, the number of events is set to the maximum allowable number.

We simulated 200 years of time series at a given vaccination coverage following a discarded initial transient period of 500 years (300 years without vaccination and 200 years with vaccination at the given coverage) to reach equilibrium. We considered vaccination coverage ranging 0–95% and birth rates corresponding to that in Ethiopia and Chad (30 and 40 births per 1000 persons per year respectively [21]), amplitude of seasonal forcing (0.3 and 0.4), and phase shift (day 0 and 180 of the year). For each setting, we divided the time series into 20 individual, non-overlapping 10-year time series and assumed that cases were observed at reporting fractions of 0.01, 0.05, 0.10, and 0.20 simulated as binomial draws from the true incidence at each time step. For each 10-year time series of observed cases, we fit the TSIR model (described in the next section) with and without correction for the double counted cases. We present the estimates of the reporting fraction, the reconstructed 10-year burden of measles, the estimated seasonal amplitude, and the estimated phase shift. Here we show the results using an annual birth rate of 40 per 1000 persons and no phase shift for 0–90% vaccination coverage. Our results hold when we extend vaccination coverage to 95%, lower the annual birth rate to 30 per 1000 persons, and add a phase shift of 180 days (refer to the Supplementary Information S1 Text for corresponding figures with these extended parameters). Code is available on Github at https://github.com/TiffanyNL/tsir.

### Fitting the TSIR model

The discrete TSIR model is described by the following equations [2, 4]:

S(t)=B(t−d)+S(t−1)−I(t), (1)

$$I(t)=p(t)I(t-1{)}^{\alpha_{1}}S(t-1{)}^{\alpha_{2}},$$ (2)

where *B*(*t*–*d*) represents births; *d* is the average duration of immunity derived from maternal antibodies; and *t* represents biweekly time steps (consistent with the infectious period for measles [2, 4]). The parameter *p*(*t*) is a time-varying transmission parameter with a 1-year (26 biweeks) period represented by

$$p(t)=\beta \left[1+\beta_{0}(1+\cos\left(2\pi t/26+\phi \right))\right].$$ (3)

The coefficients $\alpha_{1}$ and $\alpha_{2}$ are cooperativity parameters, where $\alpha_{1}=\alpha_{2}=1$ gives the standard mass-action transmission [22]. The parameters $\beta_{0}$ and *ϕ* are the amplitude and phase shift respectively.

The estimated disease burden can be obtained by fitting *I*(*t*) of the TSIR model by first estimating the reporting fraction, *r*. We start with the simulated case data and estimate *I*(*t*) through its relation with the number of reported cases *C*(*t*) by

$$I(t)=\frac{1}{r}C(t).$$ (4)

Let $S(t)=\bar{S}\;+\;Z(t)$ where the deviations *Z*(*t*) from the mean follow the same recursive relationship as *S*(*t*),

$$Z(t)=B(t-d)+Z(t-1)-\frac{1}{r}C(t).$$ (5)

Iterating through Eq 5 yields

$$Z(t)=Z_{0}+\sum_{i=1}^{t}B(i-d)-\sum_{i=1}^{t}\frac{1}{r}C(i).$$ (6)

This can be rewritten as

$$\sum_{i=1}^{t}B(i-d)=-Z_{0}+\sum_{i=1}^{t}\frac{1}{r}C(i)+Z(t)$$ (7)

which shows a simple linear regression relationship between cumulative births and cumulative cases with constant slope 1/*r*. The reporting fraction, *r*, can be estimated using the linear regression relationship between cumulative cases and cumulative births, assuming births are known through available demographic data.

Unimmunized births, *B*(*t*), are calculated as a known constant birth rate multiplied by 1–*V*, where *V* is the product of the fraction vaccinated and effectiveness, as used in other works [8, 9, 11]. Note that while we assume perfect effectiveness, these results are robust to any effectiveness as long as *V* is assumed known.

#### Proposed correction to the double-counted cases.

An individual may become infected between the time of loss of maternal immunity and vaccination and present as a case. The TSIR model would double count this individual as both an immunized birth and a case. We propose the following to correct these double-counted cases. When calculating the regression of cumulative cases against cumulative unimmunized births to estimate the reporting fraction, we subtract those cases that would be double counted. From each time step we subtract the cases that occur below the mean age of vaccination (9 months in our model), multiplied by the vaccination coverage (i.e., when coverage is low, fewer of these young cases would result in a double count). The adjusted time series of cases, C^*^(*t*), at age *a* is then


$$C^{*}(t)=C(t,a)-C(t,a<9\text{ months})\times V,$$

and the correction to Eq 4 becomes:

$$I^{*}(t)=\frac{1}{r}C^{*}(t).$$ (8)

We present the relative difference between the reporting fractions for the standard (numerator) and corrected (denominator) methods as a ratio. Thus, a value of 1 indicates that there is no difference; values <1 indicate a higher corrected estimate of *r* and corresponding higher estimate of true incidence; and values >1 indicate the opposite.

### Fitting the amplitude and phase shift

We estimated the seasonality parameters (amplitude and phase shift) by fitting the expanded transmission Eqs 2–3

$$I(t)=\beta \left[1+\beta_{0}(1+\cos\left(2\pi t/26+\phi \right))\right]I(t-1{)}^{\alpha_{1}}S(t-1{)}^{\alpha_{2}}.$$ (9)

We begin by reconstructing *S*(*t*–1) in Eq 1 by letting


$$S(t-1)=Z(t-1)+\bar{S},$$

where *Z*(*t*) represents the residuals after regressing cumulative births and cumulative cases; and $\bar{S}$ is inferred by maximizing the profile likelihood in a generalized linear model for Eq 2 without seasonality. Next we use a non-linear least squares to fit Eq 9 for five parameters: $\alpha_{1},\alpha_{2},\beta ,\beta_{0}$ and *ϕ*. The first three parameters allow non-linear dynamics, and the latter two are our estimated seasonality parameters of interest: amplitude of seasonal forcing and phase shift.

Minimum bounds are set to $\left(\alpha_{1},\alpha_{2},\beta ,\beta_{0},\phi )=(\epsilon ,\epsilon ,\epsilon ,0,0\right)$; the maximum bound for all parameters is ∞; and the initial starting point is $\left(\alpha_{1},\alpha_{2},\beta ,\beta_{0},\phi )=(1,1,\epsilon ,0.5,0\right)$.

In addition we estimate the reporting fractions under the scenario of increasing vaccination coverage (instead of a fixed coverage at equilibrium) with and without the proposed correction for double counted cases. We simulate 20 independent time series with increasing vaccination coverage from 60% to 90% over a 20-year period at an increasing coverage of 1.5% per year for birth rates ranging 10 to 80 births per thousand per year. This time series begins at 60% coverage, following a discarded initial transient period of 500 years (300 years without vaccination and 200 years with 60% vaccination coverage). For this we use only a single amplitude of 0.3 and phase shift of 0 days.

### Example using a rubella incidence dataset

In an example, we estimate the reporting fraction from a rubella incidence dataset using both the standard TSIR model and the TSIR model with corrections for double-counted cases. Monthly rubella incidence for 32 states of Mexico (including Mexico City, formerly known as the Federal District) was available between 1985 and 2007 [23, 24]. Between 1990 and 1999, yearly incidence was available by age groups of 1–10 years (<1, 1–4, 5–14, 15–24, 25–44, 45–64, 65+) [23, 24]. Monthly birth numbers for each state were obtained from [24, 25].

For each state, we create a time series of cases by adjusting the monthly rubella incidence data and assuming a generation time of approximately 14 days, resulting in two unobserved time steps for each observed monthly report [24]. The first is taken as a binomial draw of the observed monthly report at 50%; the second is taken as the difference between the monthly report and the binomial draw. We create a time series of unimmunized births by adjusting the monthly births from one observed monthly report to two unobserved reports in the same way. Following the start of vaccination against rubella with the MMR vaccine at 12 months old in 1998 [26], we calculate the unimmunized births by discounting the total births by 80%, as in [24]. We then estimate the reporting fraction using the standard TSIR model without maternal immunity (*d* = 0 in Eq 7).

We estimate the reporting fraction again with a correction to the double-counted cases using the age-structured incidence data available for 1990–1997. For each year here, we first calculate the fraction, *x*(*t*), of cases that occur in children before rubella vaccination age (12 months). Next, in the time series of cases without age structure, we estimate the number of cases occurring under 12 months of age by taking a binomial draw with probability *x*(*t*) for each corresponding year. We correct for double-counted cases by subtracting the total bi-monthly cases by the estimated cases under 12 months of age multiplied by 80% vaccination coverage. We use this ‘corrected’ time series of cases and the time series of births to estimate the reporting fraction.

## Results

**Fig 1:**
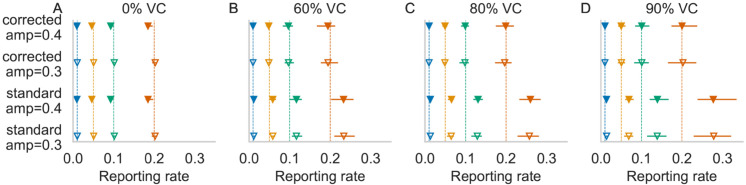
Estimated reporting fractions for varying vaccination coverage (VC) using the standard and corrected TSIR models. Colors indicate different reporting fractions; vertical lines indicate simulated truth. The mean is shown by triangles with the range (horizontal lines) of estimates from 20 simulations for amplitudes of 0.3 and 0.4.

For time series at equilibrium without vaccination, the estimated reporting fractions are unbiased for both birth rates and all combinations of true reporting fraction, amplitude, and phase shift, as expected (Fig 1A and S1 Fig A, G). At high vaccination coverage, however, there is a positive (absolute and relative) bias using the standard TSIR model fit for all combinations of reporting fractions, amplitude and phase shift (Fig 1B–1D, S1 Fig C–F, I–L). The bias is larger under a higher birth rate as a larger fraction of cases occur in young children below the age of vaccination (S1 Fig). The biggest absolute error of the estimated reporting fractions using the standard TSIR model fit is observed at 90% vaccination coverage for a true reporting fraction of 0.20, estimated 0.278 (Fig 1D, shown in red). The relative bias increases with vaccination coverage but does not change with different reporting fractions (Supplementary Information S1 Text). For example, the relative bias of the estimated reporting fraction using the standard TSIR model increases from approximately 0.01 without vaccination to approximately 0.39 at 90% vaccination coverage (for an amplitude of 0.3 across all chosen reporting fractions). Using the corrected TSIR model, the bias remains at around 0.01 across all vaccination coverage (Fig 1, S1 Text). This bias is also observed under an assortative (Kenya-like) contact mixing structure, under which violates the implicit homogeneous mixing assumption of the TSIR model (S2 Fig). For example, at 60% vaccination coverage for a true reporting fraction of 0.10, the estimated reporting fractions are 0.154 and 0.117 under assortative and homogeneous contact mixing respectively (S2 Fig). For full tables of values of estimated reporting fractions and bias using the standard and corrected TSIR models, see the Supplementary Information (S1 Text).

**Fig 2:**
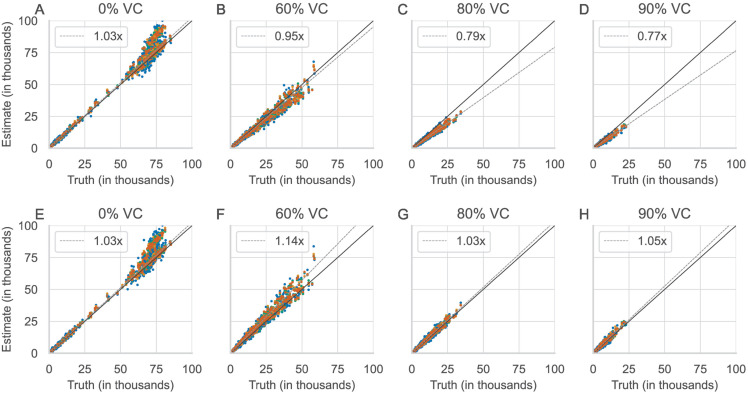
The estimated annual burden of measles for varying vaccination coverage. A–D: Using the standard TSIR estimators of the reporting fraction. E–H: Using the corrected TSIR estimators of the reporting fraction. Diagonal lines are the line of identity (solid) and the regression of the estimated and true measles burden (dashed). Colors differentiate reporting fractions (0.01, 0.05, 0.10, and 0.20). Each reporting fraction shows 80 estimates (20 annual estimates per combination of amplitude and phase shift).

The estimated annual burden of measles is plotted against the true annual burden (Fig 2), forming an elongated shape along the line of identity to indicate years of low and high measles burden as in biennial dynamics. As expected, the total annual burden decreases as vaccination coverage increases. As a consequence of the positive bias in the reporting fractions, the estimated annual burden is negatively biased (Fig 2). This bias is observed for both birth rates and all chosen reporting fractions, amplitudes, and phase shifts (S3 Fig and S4 Fig). A regression of the estimated annual burden of measles against the true annual burden of measles shows underestimation at higher vaccination coverage, with the slope of the linear regression at a minimum of 0.77 at 90% coverage (Fig 2D). At lower vaccination coverage, the burden is slightly overestimated from the truth, as observed by a slope of regression slightly above 1 (S3 Fig B and H). This is due to a slightly underestimated reporting fraction at low vaccination coverage. The estimates of annual burden using different true reporting fractions do not differ significantly.

Correcting for the double counting of cases that occurs before the age of vaccination removes the positive bias in the estimate of reporting fraction (Fig 1, S5 Fig). The biggest absolute difference of the estimated reporting fraction to true reporting fraction is now -0.03 with the correction (at 95% vaccination coverage at the higher birth rate; S5 Fig F), compared to a maximum difference of 0.078 without correction. These reporting fraction estimates align more closely to the truth for both birth rates.

Using these estimates of the reporting fraction with the proposed correction for the double counting of cases also removes the negative bias in the estimated annual burden of measles (Fig 2E–2F, S4 Fig). The regression of the estimated and true annual burden using the corrected TSIR estimator now shows an overestimation at higher vaccination coverage (Fig 2F–2H, S4 Fig). At 90% coverage with the corrected TSIR estimator, the estimated annual burden is now higher than the true annual burden by 5% on average (Fig 2H). This compares with the estimated burden using the standard TSIR estimator (without the correction), where at 90%, the estimated annual burden is smaller than the true annual burden by 23% on average (Fig 2D).

**Fig 3:**
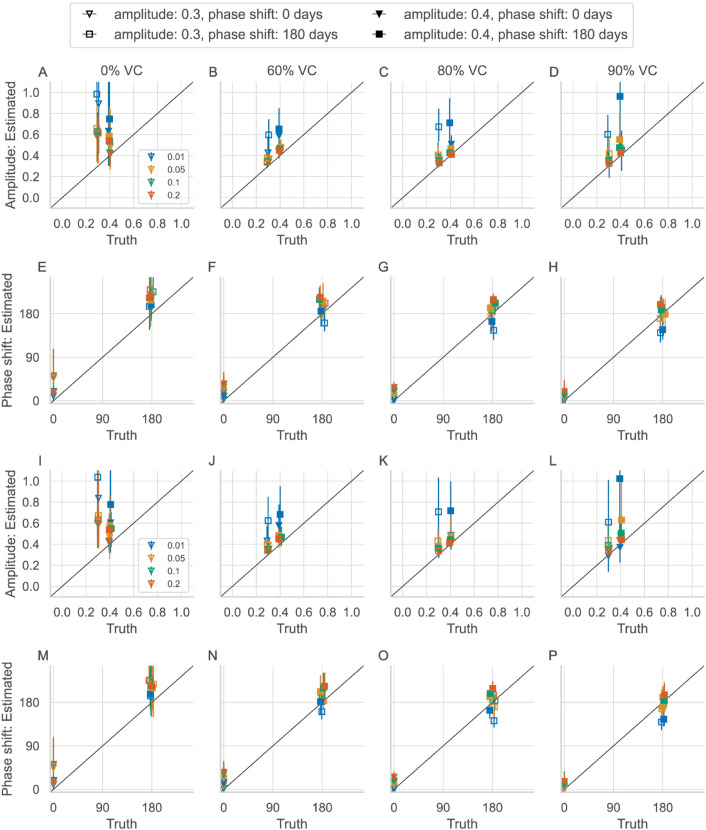
Estimated amplitude and phase shift over different vaccination coverage. A–H: Using the standard TSIR estimator of the reporting fraction. I–P: Using the corrected TSIR estimators of the reporting fraction. Each panel shows the mean (marker) and range (vertical lines) over 20 ten-year intervals. Colors differentiate the 4 chosen reporting fractions.

The estimated seasonality parameters are plotted against the true seasonality parameters using the standard TSIR estimator (Fig 3A–3H and S6 Fig) and the corrected TSIR estimator (Fig 3I–3P and S7 Fig). Despite the bias in the estimated reporting fraction and incidence, these estimates are unbiased for all 4 reporting fractions (Fig 3, S6 Fig). Using the corrected TSIR estimator also does not affect the estimate of the amplitude and phase shift (Fig 3I–3P, S7 Fig). However, the estimated amplitude is poor at 0.01 reporting fraction (in blue) due to many zeros in the reconstructed incidence. The frequency of zeros make up as much as 60% of the reconstructed time series at 0.01 reporting fraction, compared with less than 10% of the same at 0.20 reporting fraction (S8 Fig). A true reporting fraction of 0.05 and higher gives reasonably accurate estimated amplitude and range.

**Fig 4:**
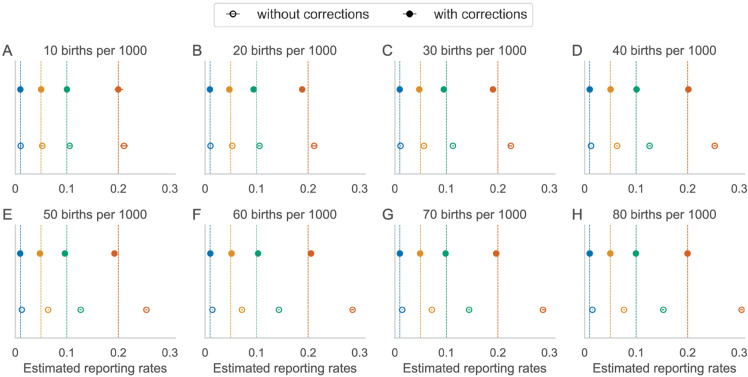
Estimated reporting fractions for simulations with increasing vaccination coverage from 60% to 90% over 20 years. Vaccination coverage increases at 1.5% per year for reporting fractions (0.01, 0.05, 0.1 and 0.2; vertical lines) and different birth rates.

Realistically, few settings in the world have long-term equilibrium dynamics at a constant level of vaccination. The bias in the estimated reporting fraction that occurs at equilibrium is also present in transient time series dynamics and increases with birth rate (Fig 4). In the transient time series, vaccination coverage is increasing from 60% to 90% over 20 years (Fig 4). For a true reporting fraction of 0.20, the estimated reporting fraction without using the proposed bias correction is 0.211 at 10 births per 1000 and grows to an estimated 0.305 at 80 births per 1000 (Fig 4). Using the proposed bias correction overcomes this bias in the estimate of the reporting fraction for all birth rates and reporting fractions (Fig 4).

As a case study, we use data of rubella incidence from Mexico (S9 Fig) and estimate the reporting fractions using the standard and corrected TSIR models (Table 1). The difference is lowest in Yucatan (0.265) and highest in Colima (4.024), reflecting a change in estimated total rubella burden that is about 4 times higher or lower, respectively.

**Table 1:** Estimated reporting fractions, r, for Mexican states. Columns are the estimated reporting fractions by the standard TSIR estimator, by the corrected TSIR estimator, and the relative difference between the standard and corrected TSIR estimator as a ratio.

State	r_standard	r_corrected	difference
Aguascalientes	0.0241	0.0243	0.992
Baja California	0.0204	0.0378	0.540
Baja California Sur	0.0477	0.0191	2.497
Campeche	0.0210	0.0221	0.950
Coahuila	0.0240	0.0443	0.542
Colima	0.0169	0.0042	4.024
Chiapas	0.0023	0.0049	0.469
Chihuahua	0.0165	0.0553	0.298
Distrito Federal	0.0251	0.0461	0.544
Durango	0.0081	0.0080	1.013
Guanajuato	0.0132	0.0082	1.610
Guerrero	0.0077	0.0072	1.069
Hidalgo	0.0079	0.0095	0.832
Jalisco	0.0111	0.0093	1.194
Mexico	0.0146	0.0205	0.712
Michoacan	0.0036	0.0056	0.643
Morelos	0.0177	0.0248	0.714
Nayarit	0.0111	0.0343	0.324
Nuevo Leon	0.0524	0.0499	1.050
Oaxaca	0.0104	0.0182	0.571
Puebla	0.0072	0.0040	1.800
Queretaro	0.0254	0.0190	1.337
Quintana Roo	0.0240	0.0264	0.909
San Luis Potosi	0.0112	0.0157	0.713
Sinaloa	0.0121	0.0142	0.852
Sonora	0.0187	0.0168	1.113
Tabasco	0.0065	0.0127	0.512
Tamaulipas	0.0392	0.1028	0.381
Tlaxcala	0.0154	0.0130	1.185
Veracruz	0.0143	0.0286	0.500
Yucatan	0.0341	0.1288	0.265
Zacatecas	0.0128	0.0121	1.058

## Discussion

We have illustrated that a standard simplifying assumption of the TSIR model, that the effect of vaccination can be represented as a reduction in the rate of susceptible recruitment, leads to a positive bias in the estimate of the reporting fraction and a negative bias in the estimate of the true incidence. When vaccination is not given immediately after birth, as is common for vaccine-preventable diseases such as measles, mumps, and rubella, then the standard assumption to the TSIR model to accommodate vaccination may lead to double counting of young cases as immunized births. The magnitude of this bias (absolute and relative) will increase when birth rates are high (i.e., there are numerically many pre-vaccine age children to become infected) and when vaccination rates are high (i.e., a larger fraction of pre-vaccine age children who become infected would likely go on to be vaccinated). We proposed a method to correct for double counting and show that the correction led to a more accurate estimation of the reporting fraction and the incidence of infection. Many studies have used the TSIR model to capture seasonal variation in time series data [2, 4, 27, 28]. We also show that the estimation of seasonality parameters was not affected by the bias in the estimate of the reporting fraction.

The TSIR model is a tractable approach to bridging dynamical epidemic models and incidence data. However, it makes several assumptions that may not be realistic for certain infections. It assumes that death from the disease is negligible, though it may become significant over a long time scale. It assumes a fixed infectious period, which is not realistic for chronic infections. It also assumes a constant reporting rate over time, although in reality it can vary. We note that the TSIR model was originally developed for application in a vaccine-free setting [2]. Thus, the bias we have identified is not a flaw of the model itself. Rather, it arises from the naive extension to settings with vaccination. Bjørnstad *et al*. [4] notes other biases that exist (e.g. estimation of the basic reproduction number) due to implicit assumptions such as homogeneous mixing. Further, many applications of the TSIR model to fit observed time series have relied on time series that are not disaggregated by pre- and post-vaccine age individuals.

Using our proposed correction for rubella time series in Mexico, we see large variability in the differences between the standard and corrected reporting rates. We were unable to determine the accuracy of the estimated reporting ratios in these data due to a lack of ‘ground truth’. However, the implication of this effect is that it could bias the estimated total rubella burden by 2 to 3 times at the state level. In addition to the bias due to incident cases in children too young to vaccinate, additional model assumptions, such as homogeneous contact mixing and constant reporting rate, may contribute to these differences.

The bias correction that we have proposed here requires the availability of age-specific case data, at least grouped as pre- and post-vaccine age. Where such data are not available, this analysis serves as a caution on interpreting the estimates of reporting fraction and incidence of infection in settings where the effect of the bias may be high.

The simplicity of the standard TSIR model and its ease of application for under-reported time series have made it an enduring tool for analysis of time series data even into the vaccine era [8, 10, 11, 29]. The bias identified here should be a cautionary note that simplifying assumptions, while often practically necessary, should be revisited as the quality and resolution of data increase.

## Supporting information

S1 Fig Estimated reporting fractions using the standard TSIR model and different birth rates. Estimated reporting fractions using the standard TSIR model for 40 (A–F) and 30 births (G–L) per 1000 persons per year and varying vaccination coverage (VC). Colors indicate different reporting fractions; vertical lines indicate simulated truth and symbols indicate mean and range (horizontal lines) of estimates from 20 simulations for different amplitudes (fill) and phase shifts (shape).
(TIF)

S2 Fig Estimated reporting fractions under homogeneous and assortative contact mixing. Estimated reporting fractions under homogeneous and assortative (Kenya-like) contact mixing consistent for different vaccination coverage (VC) and a high birth rate (40 births per thousand). Colors indicate different reporting fractions; vertical lines indicate simulated truth, and symbols indicate mean and range (horizontal lines) of estimates from 20 simulations.
(TIF)

S3 Fig Estimated annual burden of measles using the standard TSIR estimator and different birth rates. The estimated annual burden of measles using the standard TSIR estimator of the reporting fraction for 40 (A–F) and 30 (G–L) births per 1000 persons per year and vaccination coverage ranging 0–95%. Diagonal lines are the line of identity (*y* = *x*; solid) and the regression of the estimated and true measles burden (dashed). Colors differentiate reporting fractions (0.01, 0.05, 0.10, and 0.20). Each reporting fraction shows 80 estimates (20 annual estimates per combination of amplitude and phase shift).
(TIF)

S4 Fig Estimated annual measles burden using the corrected TSIR estimator and different birth rates. The estimated annual measles burden using the corrected TSIR estimator of the reporting fraction for 40 (A–F) and 30 (G–L) births per 1000 persons per year and vaccination coverage ranging 0–95%. Diagonal lines are the line of identity (*y* = *x*; solid) and the regression of the estimated and true measles burden (dashed). Colors differentiate reporting fractions (0.01, 0.05, 0.10, and 0.20). Each reporting fraction shows 80 estimates (20 annual estimates per combination of amplitude and phase shift).
(TIF)

S5 Fig Estimated reporting fractions using the corrected TSIR estimator and different birth rates. Estimated reporting fractions using the corrected TSIR estimator of the reporting fraction for 40 (A–F) and 30 (G–L) births per 1000 persons per year for vaccination coverage (VC) ranging 0–95%. Colors indicate different reporting fractions; vertical lines indicate simulated truth and symbols indicate mean and range (horizontal lines) of estimates from 20 simulations for different amplitudes (fill) and phase shifts (shape).
(TIF)

S6 Fig Estimated amplitude and phase shift using the standard TSIR estimator and different birth rates. Estimated amplitude and phase shift using the standard TSIR estimator of the reporting fraction for 40 (A–L) and 30 (M–X) births per 1000 persons per year and vaccination coverage ranging 0–95%. Each panel shows the mean (marker) and range (vertical lines) over 20 ten-year intervals. Colors differentiate the 4 chosen reporting fractions.
(TIF)

S7 Fig Estimated amplitude and phase shift using the corrected TSIR estimator and different birth rates. Estimated amplitude and phase shift using the corrected TSIR estimator of the reporting fraction for 40 (A–L) and 30 (M–X) births per 1000 persons per year and vaccination coverage ranging 0–95%. Each panel shows the mean (marker) and range (vertical lines) over 20 ten-year intervals. Colors differentiate the 4 chosen reporting fractions.
(TIF)

S8 Fig Frequency of zeroes in the reconstructed time series. Frequency of zeroes in the reconstructed time series of incidence for different estimated reporting fraction and estimated amplitude for 20 ten-year time series. True reporting fractions are 0.01 (blue), 0.05 (yellow), 0.10 (green), and 0.20 (red). Here the true amplitude is 0.3 with 0 phase shift.
(TIF)

S9 Fig Time series of rubella in Mexico. Time series of half-monthly rubella cases from 32 states in Mexico. The red line indicates the start of vaccination.
(TIF)

S1 Text Supplementary details on model structure and tables. (PDF)
