# Supplementary figures and images for: Bias in the estimated reporting fraction due to vaccination in the time-series SIR model
Source: PLoS One. 2025 Aug 22;20(8):e0330568. doi: 10.1371/journal.pone.0330568 (PMC12373160; doi:10.1371/journal.pone.0330568)

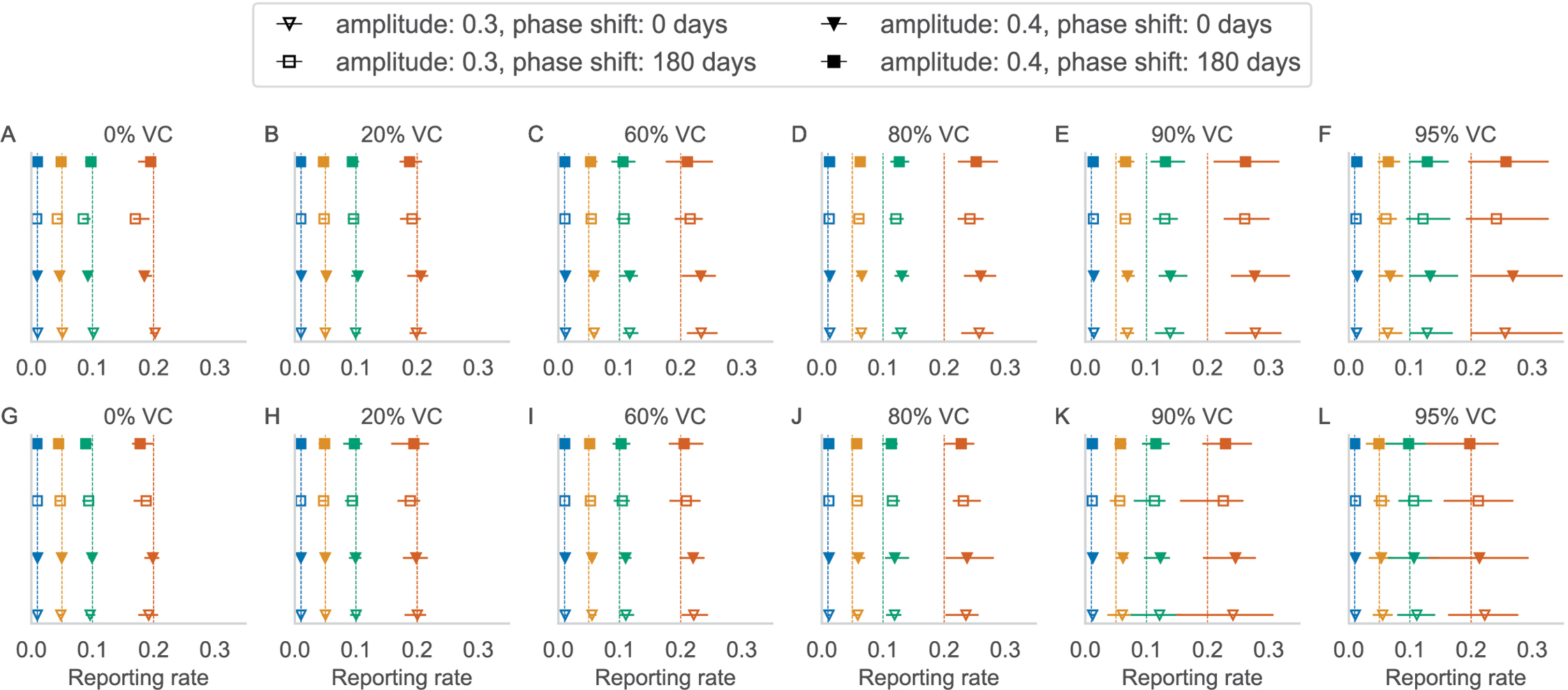

Supplement: S1 Fig — Estimated reporting fractions using the standard TSIR model for 40 (A–F) and 30 births (G–L) per 1000 persons per year and varying vaccination coverage (VC). Colors indicate different reporting fractions; vertical lines indicate simulated truth and symbols indicate mean and range (horizontal lines) of estimates from 20 simulations for different amplitudes (fill) and phase shifts (shape). (TIF) [file pone.0330568.s001.tif]

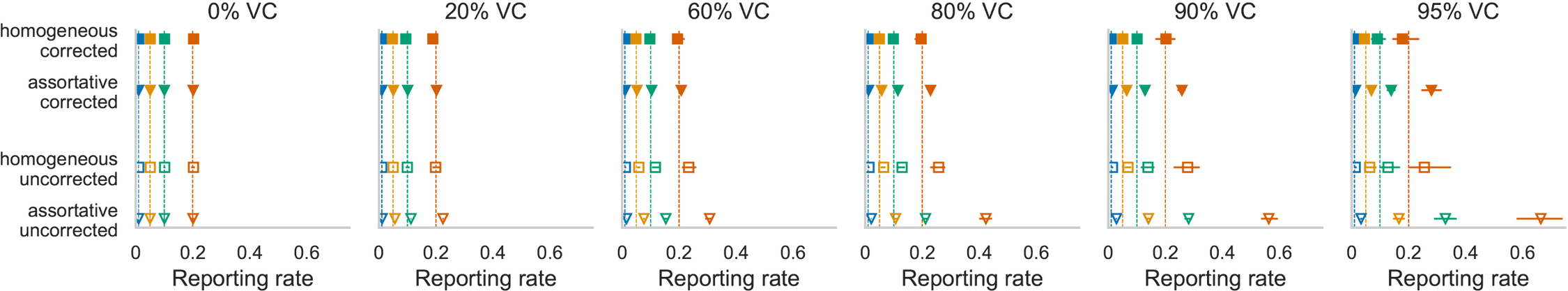

Supplement: S2 Fig — Estimated reporting fractions under homogeneous and assortative (Kenya-like) contact mixing consistent for different vaccination coverage (VC) and a high birth rate (40 births per thousand). Colors indicate different reporting fractions; vertical lines indicate simulated truth, and symbols indicate mean and range (horizontal lines) of estimates from 20 simulations. (TIF) [file pone.0330568.s002.tif]

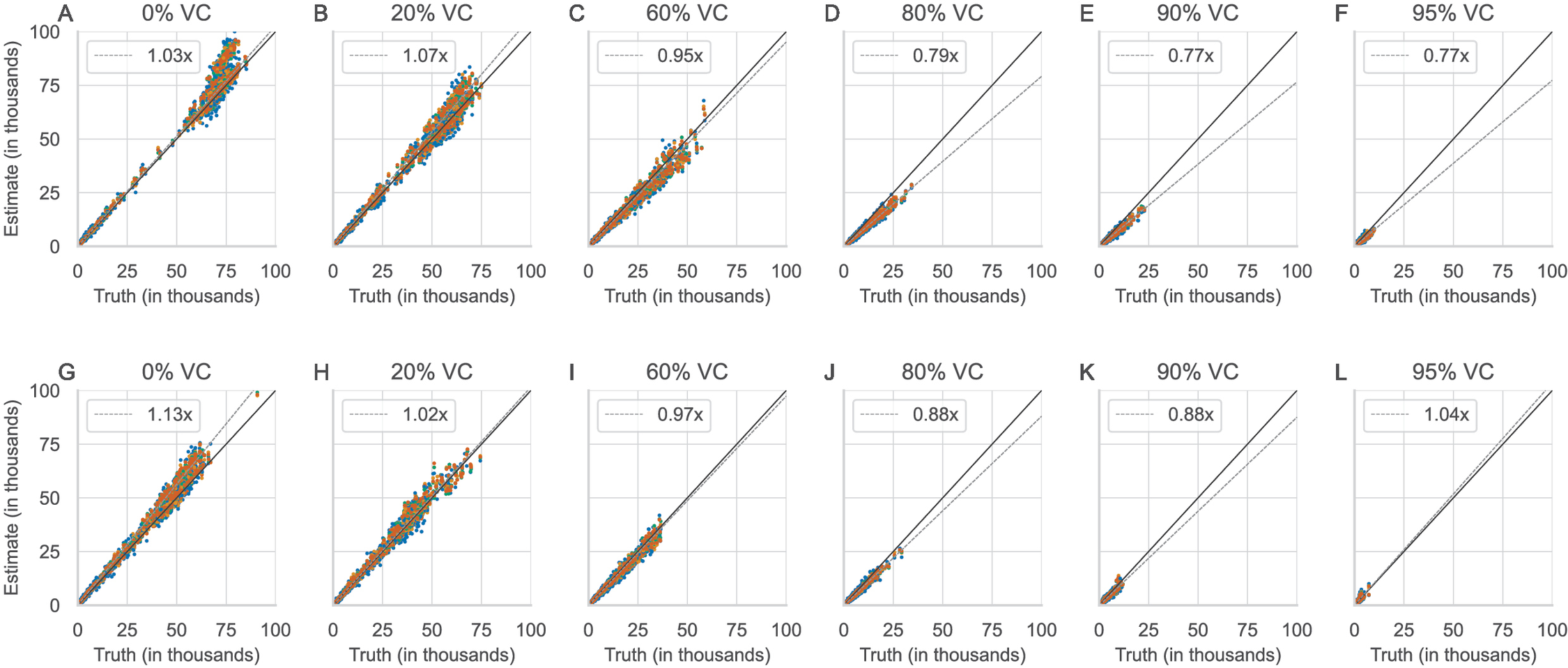

Supplement: S3 Fig — The estimated annual burden of measles using the standard TSIR estimator of the reporting fraction for 40 (A–F) and 30 (G–L) births per 1000 persons per year and vaccination coverage ranging 0–95%. Diagonal lines are the line of identity (y = x; solid) and the regression of the estimated and true measles burden (dashed). Colors differentiate reporting fractions (0.01, 0.05, 0.10, and 0.20). Each reporting fraction shows 80 estimates (20 annual estimates per combination of amplitude and phase shift). (TIF) [file pone.0330568.s003.tif]

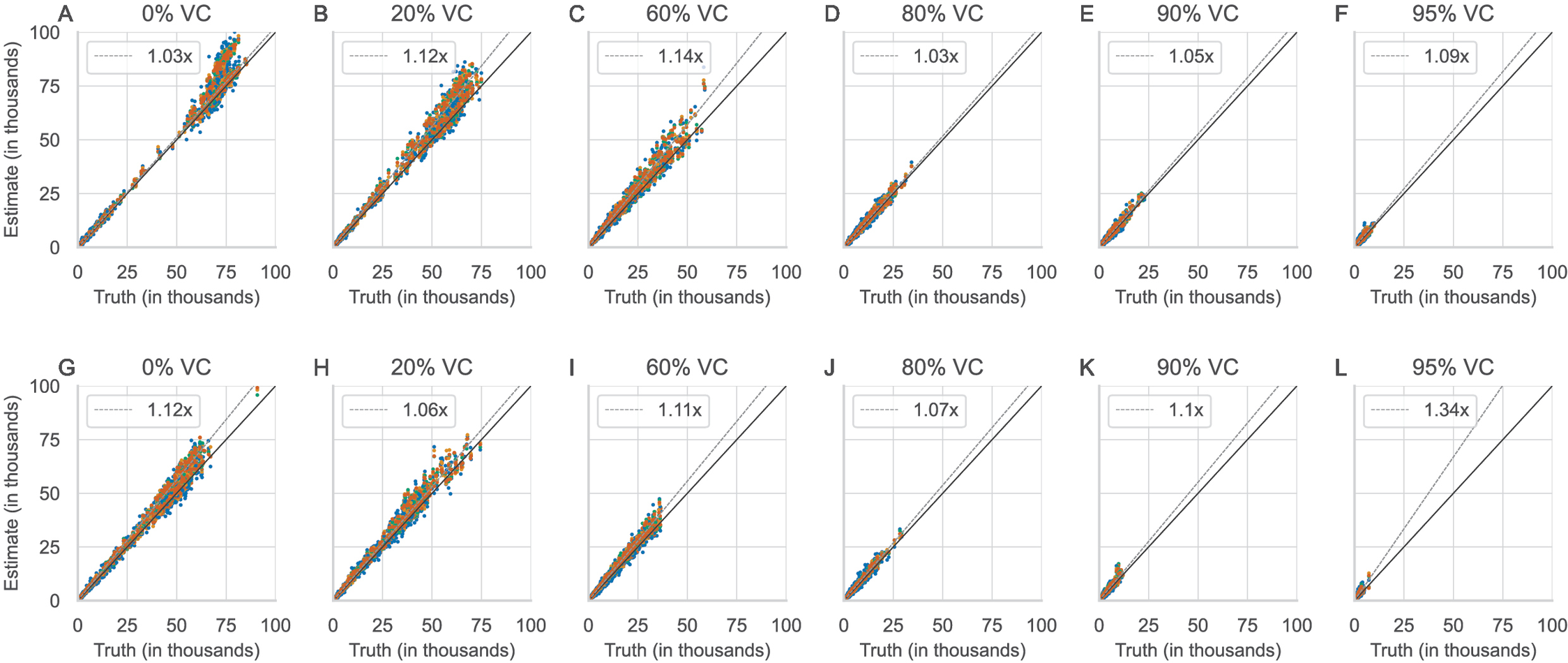

Supplement: S4 Fig — The estimated annual measles burden using the corrected TSIR estimator of the reporting fraction for 40 (A–F) and 30 (G–L) births per 1000 persons per year and vaccination coverage ranging 0–95%. Diagonal lines are the line of identity (y = x; solid) and the regression of the estimated and true measles burden (dashed). Colors differentiate reporting fractions (0.01, 0.05, 0.10, and 0.20). Each reporting fraction shows 80 estimates (20 annual estimates per combination of amplitude and phase shift). (TIF) [file pone.0330568.s004.tif]

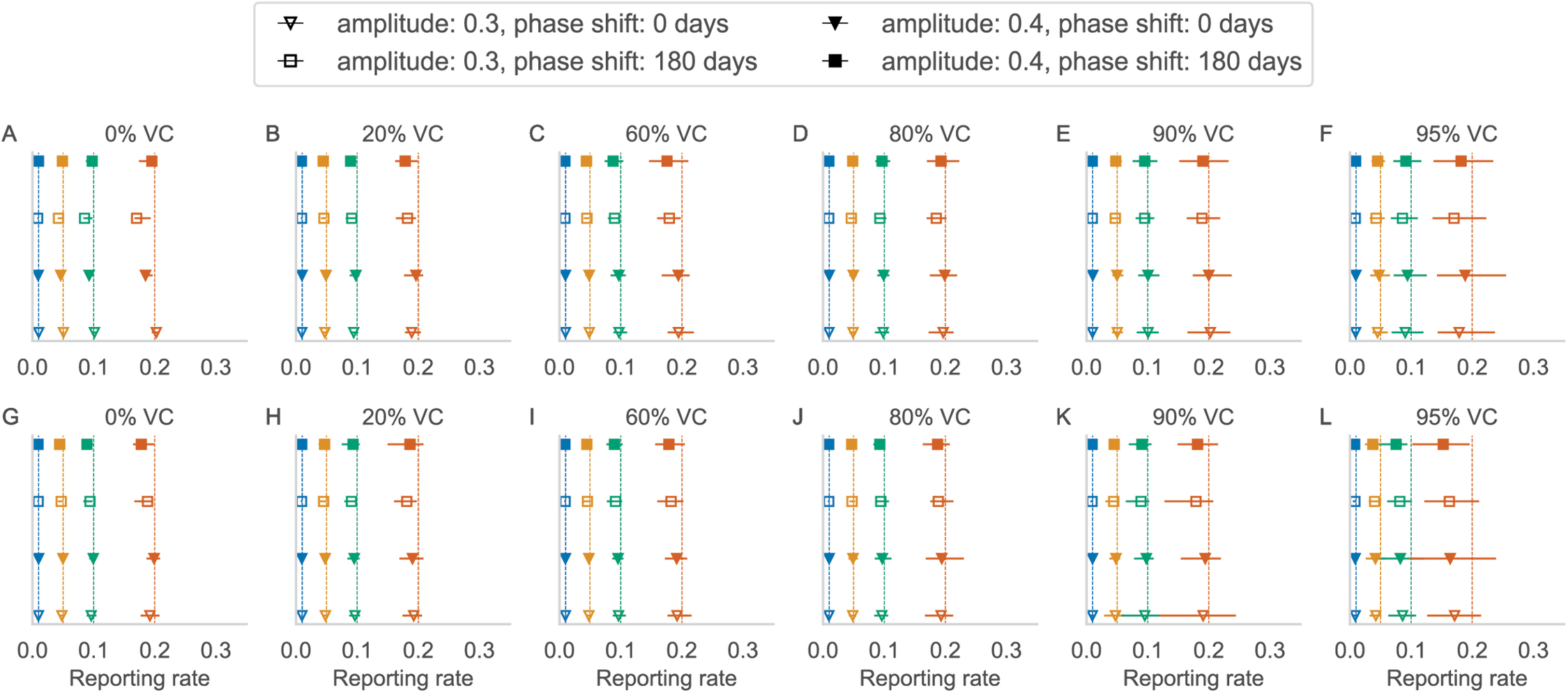

Supplement: S5 Fig — Estimated reporting fractions using the corrected TSIR estimator of the reporting fraction for 40 (A–F) and 30 (G–L) births per 1000 persons per year for vaccination coverage (VC) ranging 0–95%. Colors indicate different reporting fractions; vertical lines indicate simulated truth and symbols indicate mean and range (horizontal lines) of estimates from 20 simulations for different amplitudes (fill) and phase shifts (shape). (TIF) [file pone.0330568.s005.tif]

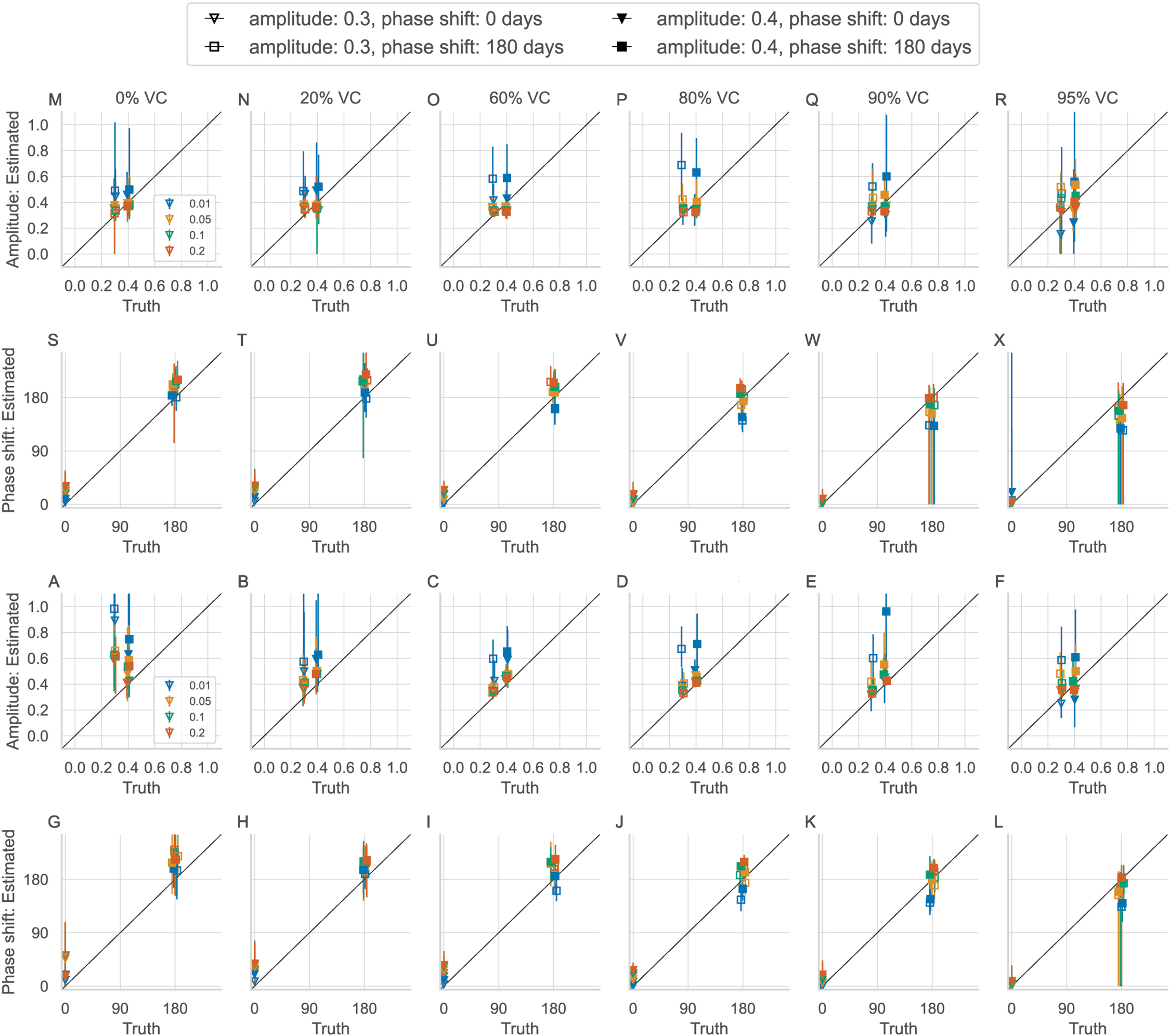

Supplement: S6 Fig — Estimated amplitude and phase shift using the standard TSIR estimator of the reporting fraction for 40 (A–L) and 30 (M–X) births per 1000 persons per year and vaccination coverage ranging 0–95%. Each panel shows the mean (marker) and range (vertical lines) over 20 ten-year intervals. Colors differentiate the 4 chosen reporting fractions. (TIF) [file pone.0330568.s006.tif]

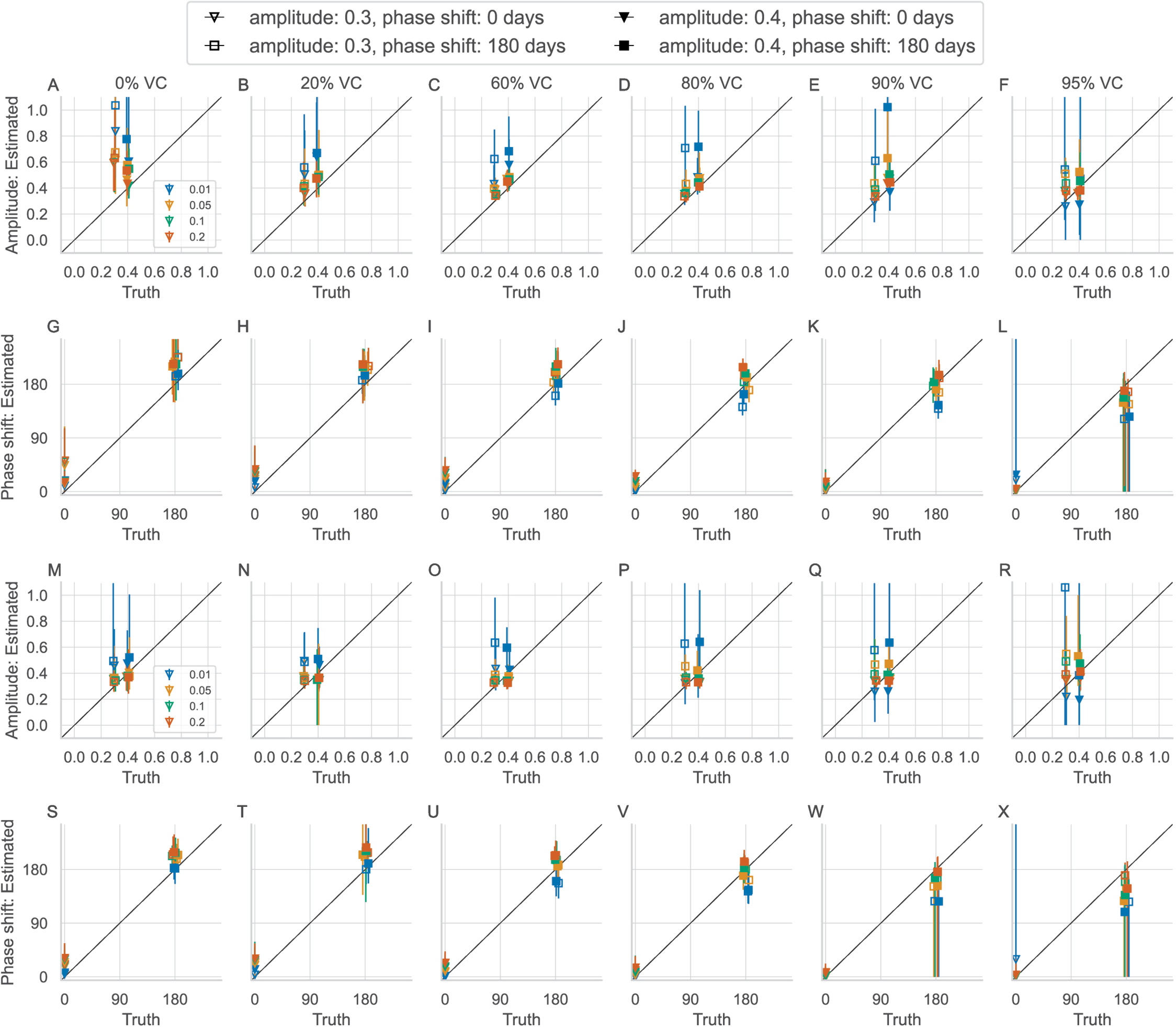

Supplement: S7 Fig — Estimated amplitude and phase shift using the corrected TSIR estimator of the reporting fraction for 40 (A–L) and 30 (M–X) births per 1000 persons per year and vaccination coverage ranging 0–95%. Each panel shows the mean (marker) and range (vertical lines) over 20 ten-year intervals. Colors differentiate the 4 chosen reporting fractions. (TIF) [file pone.0330568.s007.tif]

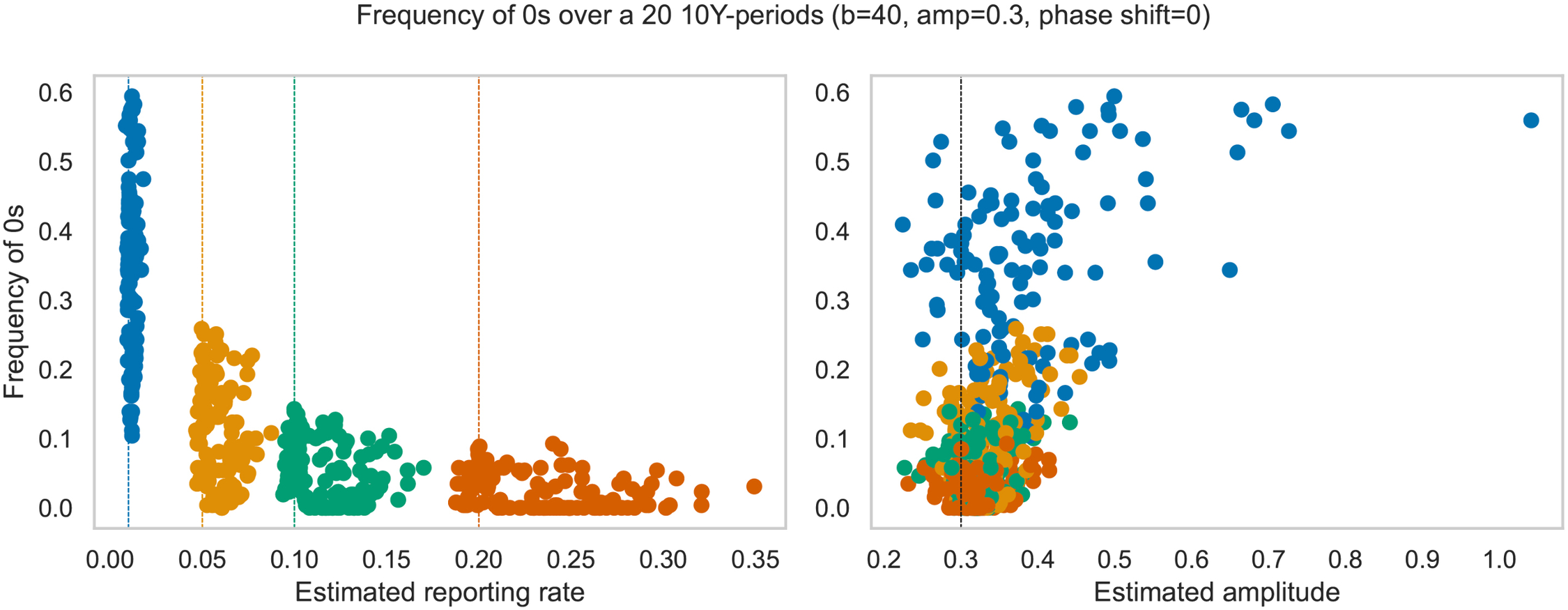

Supplement: S8 Fig — Frequency of zeroes in the reconstructed time series of incidence for different estimated reporting fraction and estimated amplitude for 20 ten-year time series. True reporting fractions are 0.01 (blue), 0.05 (yellow), 0.10 (green), and 0.20 (red). Here the true amplitude is 0.3 with 0 phase shift. (TIF) [file pone.0330568.s008.tif]

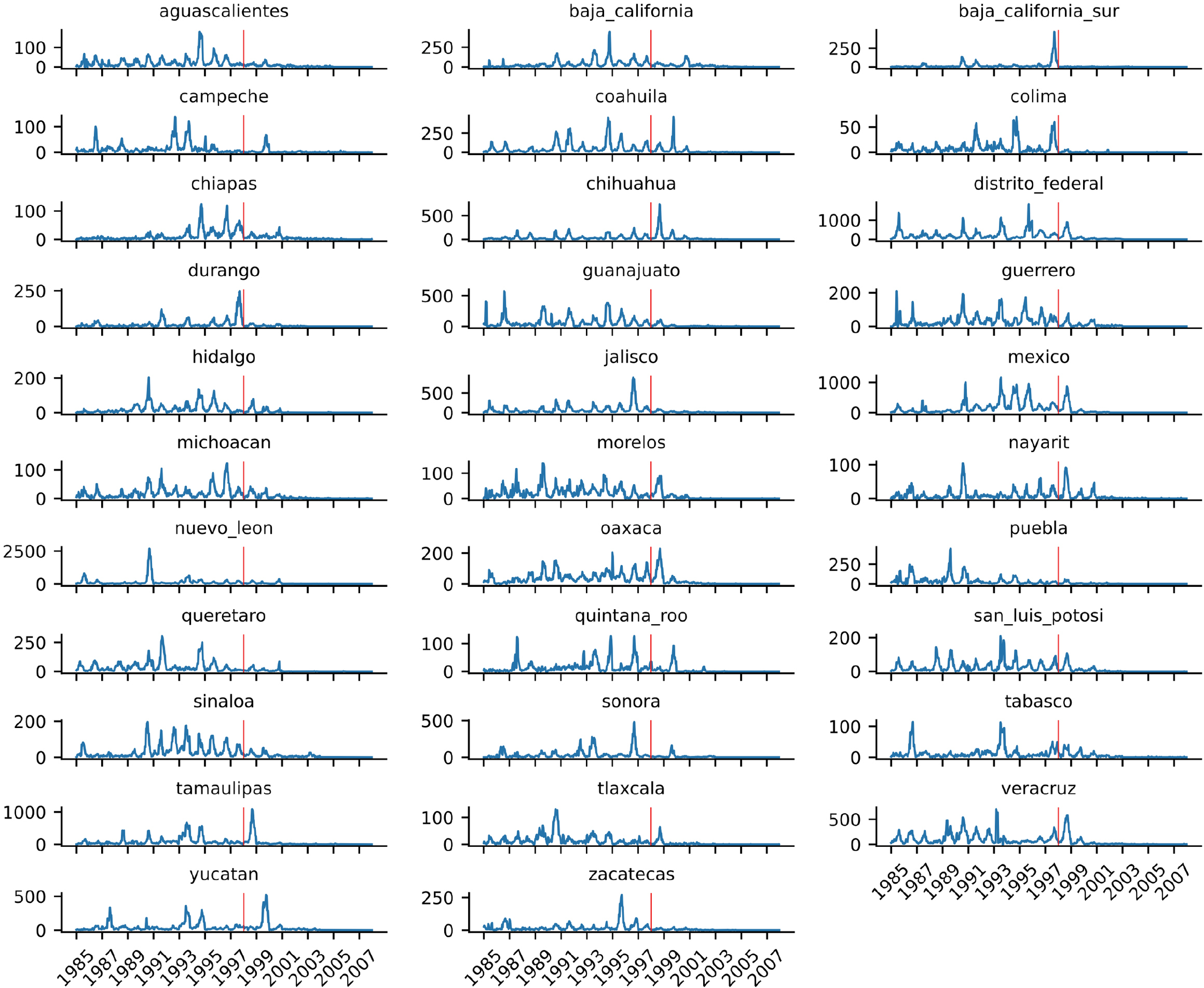

Supplement: S9 Fig — Time series of half-monthly rubella cases from 32 states in Mexico. The red line indicates the start of vaccination. (TIF) [file pone.0330568.s009.tif]
