# Supplementary material for: Bias in the estimated reporting fraction due to vaccination in the time-series SIR model
Source: PLoS One. 2025 Aug 22;20(8):e0330568. doi: 10.1371/journal.pone.0330568 (PMC12373160; doi:10.1371/journal.pone.0330568)
Supplement: S1 Text — (PDF) [file pone.0330568.s010.pdf]

# Supplementary Information for: Bias in the estimated reporting fraction due to vaccination in the time-series SIR model

Tiffany Leung[1], Matthew Ferrari[1*]
[1]Center for Infectious Disease Dynamics, Pennsylvania State University, University Park, PA, USA

*Corresponding author: mjf283@psu.edu

## Model description and equations

We used an age-structured discrete-time stochastic transmission model that incorporates both epidemiological and demographic transitions, building on a framework introduced by Klepac and Caswell [1] and Klepac et al. [2]. We describe the model structure following the notation of Metcalf et al. [3] and Winter et al. [4]. The model population is structured into an unvaccinated group and a vaccinated group. Individuals within each vaccination status group are further divided in epidemiological classes (temporarily immune with maternal antibodies, $M, M_v$; susceptible, $S, S_v$; infectious, $I, I_v$; recovered $R, R_v$) where the unvaccinated group is without subscript, and the vaccinated group is denoted by subscript $v$. The population is stratified into 40 discrete age classes (24 monthly classes from ages 0 to 2 years; 1 class from age 2 to 5 years; 14 5-year classes from age 5 to 75 years; and 1 class of age 75 years and older). At every time step a large transition matrix defines transitions from every possible epidemiological stage and age class combination to every other possible epidemiological stage and age class combination.

The transition matrix is described in two steps. First ignoring demography (aging and survival), we define matrix $\mathbf{A}_{a,t}$, which describes the epidemiological transitions within each age class $a$ and discrete time-step $t$ (set to 1 day), as

$$\mathbf{A}_{a,t} = \begin{bmatrix} (1-d_a)(1-\nu_a) & 0 & 0 & 0 & 0 & 0 & 0 & 0 \\ d_a(1-\nu_a) & 1-\phi_a(\mathbf{n}(t))(1-\nu_a) & 0 & 0 & 0 & 0 & 0 & 0 \\ 0 & \phi_a(\mathbf{n}(t))(1-\nu_a) & 1-\gamma & 0 & 0 & 0 & 0 & 0 \\ 0 & 0 & \gamma & 1-\nu_a & 0 & 0 & 0 & 0 \\ \nu_a & 0 & 0 & 0 & 1-d_a & 0 & 0 & 0 \\ 0 & \nu_a(1-\mathrm{VE}) & 0 & 0 & d_a & 1-\phi(\mathbf{n}(t)) & 0 & 0 \\ 0 & 0 & 0 & 0 & 0 & \phi_a(\mathbf{n}(t)) & 1-\gamma & 0 \\ 0 & \nu_a\mathrm{VE} & 0 & \nu_a & 0 & 0 & \gamma & 1 \end{bmatrix}.$$

The eight rows and columns represent the stages $M, S, I, R, M_v, S_v, I_v, R_v$, respectively. In the transition matrix, $d_a$ is the probability an individual in age class $a$ loses maternal immunity; $\phi_a$ is the probability an individual in age class $a$ becomes infected; $\gamma$ is the probability of recovery; and $\nu_a$ is the probability an individual in age class $a$ is vaccinated. A susceptible individual is successfully vaccinated with probability VE, reflecting the vaccine efficacy. Vaccination has no effect on individuals with maternal immunity or recovered individuals. The infection probability $\phi$ (also called the force of infection) is a function of $\mathbf{n}(t)$, a vector describing the population at time $t$

$$\mathbf{n}(t) = (M_{1,t}, S_{1,t}, I_{1,t}, R_{1,t}, M_{v,1,t}, S_{v,1,t}, I_{v,1,t}, R_{v,1,t}, M_{2,t}, ..., R_{v,z,t})^T$$

according to

$$\phi(\mathbf{n}(t)) = 1 - \exp\left[-\sum_{j}^{z} \beta_{a,j,t}(I_{j,t} + I_{v,j,t}) \Big/ \sum \mathbf{n}(t)\right]$$

where $z$ is the total number of age classes ($z = 40$ here); $\beta_{a,j,t}$ is the transmission rate between individuals in age class $a$ and $j$ at time-step $t$; and $I_{j,t} + I_{v,j,t}$ is the number of infected individuals in age class $j$ at time-step $t$.

Transmission to individuals in age class $a$ from individuals in age class $j$ at time $t$ is defined by $\beta_{a,j,t} = \beta c_{a,j}\left(1 + \beta_0 \cos\left(2\pi t/365\right)\right)$, where $\beta$ is the transmission coefficient calculated using the next-generation matrix ; $c_{i,j}$ is the mean contact rate between individuals in age class $j$ to $i$; and $\beta_0$ is the relative amplitude of seasonal fluctuations. The transmission coefficient was calculated with the next-generation matrix using a basic reproduction number $R_0$ of 15 [5, 6] and an average infectious period of 14 days [7, 8].

Second, we define the full transition matrix, $\mathbf{A}(\mathbf{n}(t))$, that includes both epidemiological transitions from matrix $\mathbf{A}_{a,t}$ and demographic transitions (aging and survival). This matrix is used to project the entire population forward via aging, mortality, and infection dynamics according to:

$$\mathbf{A}(\mathbf{n}(t)) = \begin{bmatrix} s_1(1-u_1)\mathbf{A}_{1,t} & 0 & 0 & \dots & 0 \\ s_1 u_1 \mathbf{A}_{1,t} & s_2(1-u_2)\mathbf{A}_{2,t} & 0 & \dots & 0 \\ 0 & s_2 u_2 \mathbf{A}_{2,t} & s_3(1-u_3)\mathbf{A}_{3,t} & \dots & 0 \\ \dots & \dots & \dots & \dots & 0 \\ 0 & 0 & 0 & \dots & s_z \mathbf{A}_{z,t} \end{bmatrix}$$

where $s_a$ is the probability that an individual in age class $a$ survives; $u_a$ is the rate of aging out of age class $a$; and $\mathbf{A}_{1,t}$, $\mathbf{A}_{2,t}$, etc., are as defined in matrix $\mathbf{A}_{a,t}$. The dynamics of the total population are then projected forward in time, such that

$$\mathbf{n}(t+1) = \mathbf{A}(\mathbf{n}(t))\mathbf{n}(t) + \mathbf{B_t}$$

where $\mathbf{B_t}$ is a vector representing the number of births at time $t$, defined as,

$$\mathbf{B_t} = (b_t, 0, 0, \dots, 0)^T \, .$$

## Supplementary tables

Here we present the tables of estimated reporting fractions using the standard TSIR model and the corrected TSIR model. Each column shows a different vaccination coverage ranging 0 to 95%, and the rows represent the true reporting fractions between 1 to 20%. We show the average (mean) over 20 simulations, while we vary the following:

- two amplitudes (0.3 and 0.4)
- two phase shifts (0 and 180 days)
- two birth rates (30 and 40 per 1000 persons per year).

The relative bias (calculated by the difference between the estimated and true reporting fraction as numerator, divided by the true reporting fraction) is shown in parentheses.

Table 1: Estimated reporting fractions using the standard TSIR model with varying vaccination coverage (0 to 95%) and true reporting fractions (0.01 to 0.20) for phase shifts (0 and 180 days) and amplitudes (0.3 and 0.4) for a birth rate of 40 per 1000 persons. The numbers are the mean of estimates from 20 simulations.

| | | **Vaccination coverage** | | | | | |
|---|---|---|---|---|---|---|---|
| | | 0 | 0.2 | 0.6 | 0.8 | 0.9 | 0.95 |
| Phase shift = 0; Amplitude = 0.3 | **r=0.01** | 0.01 (0) | 0.01 (0) | 0.012 (0.2) | 0.013 (0.3) | 0.014 (0.4) | 0.013 (0.3) |
| | **r=0.05** | 0.051 (0.02) | 0.05 (0) | 0.058 (0.16) | 0.065 (0.3) | 0.069 (0.38) | 0.064 (0.28) |
| | **r=0.10** | 0.101 (0.01) | 0.099 (-0.01) | 0.117 (0.17) | 0.129 (0.29) | 0.139 (0.39) | 0.128 (0.28) |
| | **r=0.20** | 0.202 (0.01) | 0.199 (-0.005) | 0.234 (0.17) | 0.257 (0.285) | 0.278 (0.39) | 0.256 (0.28) |
| Phase shift = 0; Amplitude = 0.4 | **r=0.01** | 0.009 (-0.1) | 0.01 (0) | 0.012 (0.2) | 0.013 (0.3) | 0.014 (0.4) | 0.014 (0.4) |
| | **r=0.05** | 0.046 (-0.08) | 0.052 (0.04) | 0.058 (0.16) | 0.065 (0.3) | 0.069 (0.38) | 0.068 (0.36) |
| | **r=0.10** | 0.092 (-0.08) | 0.103 (0.03) | 0.117 (0.17) | 0.131 (0.31) | 0.139 (0.39) | 0.133 (0.33) |
| | **r=0.20** | 0.185 (-0.075) | 0.206 (0.03) | 0.233 (0.165) | 0.26 (0.3) | 0.277 (0.385) | 0.268 (0.34) |
| Phase shift = 180; Amplitude = 0.3 | **r=0.01** | 0.008 (-0.2) | 0.01 (0) | 0.011 (0.1) | 0.012 (0.2) | 0.013 (0.3) | 0.012 (0.2) |
| | **r=0.05** | 0.042 (-0.16) | 0.048 (-0.04) | 0.054 (0.08) | 0.061 (0.22) | 0.065 (0.3) | 0.061 (0.22) |
| | **r=0.10** | 0.085 (-0.15) | 0.096 (-0.04) | 0.107 (0.07) | 0.121 (0.21) | 0.13 (0.3) | 0.121 (0.21) |
| | **r=0.20** | 0.17 (-0.15) | 0.192 (-0.04) | 0.216 (0.08) | 0.242 (0.21) | 0.261 (0.305) | 0.241 (0.205) |
| Phase shift = 180; Amplitude = 0.4 | **r=0.01** | 0.01 (0) | 0.009 (-0.1) | 0.011 (0.1) | 0.013 (0.3) | 0.013 (0.3) | 0.013 (0.3) |
| | **r=0.05** | 0.049 (-0.02) | 0.047 (-0.06) | 0.053 (0.06) | 0.063 (0.26) | 0.066 (0.32) | 0.065 (0.3) |
| | **r=0.10** | 0.097 (-0.03) | 0.094 (-0.06) | 0.106 (0.06) | 0.127 (0.27) | 0.131 (0.31) | 0.128 (0.28) |
| | **r=0.20** | 0.195 (-0.025) | 0.188 (-0.06) | 0.211 (0.055) | 0.252 (0.26) | 0.262 (0.31) | 0.257 (0.285) |

Table 2: Estimated reporting fractions using the corrected TSIR model with varying vaccination coverage (0 to 95%) and true reporting fractions (0.01 to 0.20) for phase shifts (0 and 180 days) and amplitudes (0.3 and 0.4) for a birth rate of 40 per 1000 persons. The numbers are the mean of estimates from 20 simulations.

| | | **Vaccination coverage** | | | | | |
|---|---|---|---|---|---|---|---|
| | | 0 | 0.2 | 0.6 | 0.8 | 0.9 | 0.95 |
| Phase shift = 0; Amplitude = 0.3 | **r=0.01** | 0.01 (0) | 0.009 (-0.1) | 0.01 (0) | 0.01 (0) | 0.01 (0) | 0.009 (-0.1) |
| | **r=0.05** | 0.051 (0.02) | 0.047 (-0.06) | 0.049 (-0.02) | 0.049 (-0.02) | 0.05 (0) | 0.045 (-0.1) |
| | **r=0.10** | 0.101 (0.01) | 0.094 (-0.06) | 0.097 (-0.03) | 0.098 (-0.02) | 0.101 (0.01) | 0.09 (-0.1) |
| | **r=0.20** | 0.202 (0.01) | 0.189 (-0.055) | 0.195 (-0.025) | 0.197 (-0.015) | 0.202 (0.01) | 0.178 (-0.11) |
| Phase shift = 0; Amplitude = 0.4 | **r=0.01** | 0.009 (-0.1) | 0.01 (0) | 0.01 (0) | 0.01 (0) | 0.01 (0) | 0.009 (-0.1) |
| | **r=0.05** | 0.046 (-0.08) | 0.049 (-0.02) | 0.048 (-0.04) | 0.05 (0) | 0.05 (0) | 0.047 (-0.06) |
| | **r=0.10** | 0.093 (-0.07) | 0.098 (-0.02) | 0.097 (-0.03) | 0.099 (-0.01) | 0.101 (0.01) | 0.094 (-0.06) |
| | **r=0.20** | 0.185 (-0.075) | 0.196 (-0.02) | 0.194 (-0.03) | 0.199 (-0.005) | 0.2 (0) | 0.188 (-0.06) |
| Phase shift = 180; Amplitude = 0.3 | **r=0.01** | 0.008 (-0.2) | 0.009 (-0.1) | 0.009 (-0.1) | 0.009 (-0.1) | 0.009 (-0.1) | 0.008 (-0.2) |
| | **r=0.05** | 0.042 (-0.16) | 0.046 (-0.08) | 0.045 (-0.1) | 0.046 (-0.08) | 0.047 (-0.06) | 0.043 (-0.14) |
| | **r=0.10** | 0.085 (-0.15) | 0.091 (-0.09) | 0.09 (-0.1) | 0.093 (-0.07) | 0.095 (-0.05) | 0.086 (-0.14) |
| | **r=0.20** | 0.17 (-0.15) | 0.182 (-0.09) | 0.18 (-0.1) | 0.185 (-0.075) | 0.189 (-0.055) | 0.17 (-0.15) |
| Phase shift = 180; Amplitude = 0.4 | **r=0.01** | 0.01 (0) | 0.009 (-0.1) | 0.009 (-0.1) | 0.01 (0) | 0.01 (0) | 0.009 (-0.1) |
| | **r=0.05** | 0.049 (-0.02) | 0.045 (-0.1) | 0.044 (-0.12) | 0.049 (-0.02) | 0.048 (-0.04) | 0.045 (-0.1) |
| | **r=0.10** | 0.098 (-0.02) | 0.089 (-0.11) | 0.088 (-0.12) | 0.096 (-0.04) | 0.095 (-0.05) | 0.091 (-0.09) |
| | **r=0.20** | 0.195 (-0.025) | 0.179 (-0.105) | 0.176 (-0.12) | 0.193 (-0.035) | 0.19 (-0.05) | 0.182 (-0.09) |

Table 3: Estimated reporting fractions using the standard TSIR model with varying vaccination coverage (0 to 95%) and true reporting fractions (0.01 to 0.20) for phase shifts (0 and 180 days) and amplitudes (0.3 and 0.4) for a birth rate of 30 per 1000 persons. The numbers are the mean of estimates from 20 simulations.

| | | **Vaccination coverage** | | | | | |
|---|---|---|---|---|---|---|---|
| | | 0 | 0.2 | 0.6 | 0.8 | 0.9 | 0.95 |
| Phase shift = 0; Amplitude = 0.3 | **r=0.01** | 0.01 (0) | 0.01 (0) | 0.011 (0.1) | 0.012 (0.2) | 0.012 (0.2) | 0.011 (0.1) |
| | **r=0.05** | 0.048 (-0.04) | 0.05 (0) | 0.055 (0.1) | 0.059 (0.18) | 0.06 (0.2) | 0.055 (0.1) |
| | **r=0.10** | 0.096 (-0.04) | 0.1 (0) | 0.11 (0.1) | 0.119 (0.19) | 0.121 (0.21) | 0.111 (0.11) |
| | **r=0.20** | 0.192 (-0.04) | 0.2 (0) | 0.221 (0.105) | 0.236 (0.18) | 0.242 (0.21) | 0.222 (0.11) |
| Phase shift = 0; Amplitude = 0.4 | **r=0.01** | 0.01 (0) | 0.01 (0) | 0.011 (0.1) | 0.012 (0.2) | 0.012 (0.2) | 0.011 (0.1) |
| | **r=0.05** | 0.05 (0) | 0.05 (0) | 0.055 (0.1) | 0.06 (0.2) | 0.062 (0.24) | 0.053 (0.06) |
| | **r=0.10** | 0.099 (-0.01) | 0.099 (-0.01) | 0.11 (0.1) | 0.119 (0.19) | 0.123 (0.23) | 0.107 (0.07) |
| | **r=0.20** | 0.199 (-0.005) | 0.198 (-0.01) | 0.22 (0.1) | 0.238 (0.19) | 0.246 (0.23) | 0.214 (0.07) |
| Phase shift = 180; Amplitude = 0.3 | **r=0.01** | 0.009 (-0.1) | 0.009 (-0.1) | 0.01 (0) | 0.012 (0.2) | 0.011 (0.1) | 0.011 (0.1) |
| | **r=0.05** | 0.047 (-0.06) | 0.047 (-0.06) | 0.053 (0.06) | 0.058 (0.16) | 0.056 (0.12) | 0.053 (0.06) |
| | **r=0.10** | 0.094 (-0.06) | 0.094 (-0.06) | 0.105 (0.05) | 0.115 (0.15) | 0.113 (0.13) | 0.106 (0.06) |
| | **r=0.20** | 0.188 (-0.06) | 0.189 (-0.055) | 0.209 (0.045) | 0.232 (0.16) | 0.226 (0.13) | 0.212 (0.06) |
| Phase shift = 180; Amplitude = 0.4 | **r=0.01** | 0.009 (-0.1) | 0.01 (0) | 0.01 (0) | 0.012 (0.2) | 0.011 (0.1) | 0.01 (0) |
| | **r=0.05** | 0.045 (-0.1) | 0.049 (-0.02) | 0.051 (0.02) | 0.057 (0.14) | 0.057 (0.14) | 0.049 (-0.02) |
| | **r=0.10** | 0.089 (-0.11) | 0.097 (-0.03) | 0.103 (0.03) | 0.114 (0.14) | 0.115 (0.15) | 0.098 (-0.02) |
| | **r=0.20** | 0.178 (-0.11) | 0.195 (-0.025) | 0.206 (0.03) | 0.228 (0.14) | 0.229 (0.145) | 0.198 (-0.01) |

Table 4: Estimated reporting fractions using the corrected TSIR model with varying vaccination coverage (0 to 95%) and true reporting fractions (0.01 to 0.20) for phase shifts (0 and 180 days) and amplitudes (0.3 and 0.4) for a birth rate of 30 per 1000 persons. The numbers are the mean of estimates from 20 simulations.

| | | **Vaccination coverage** | | | | | |
|---|---|---|---|---|---|---|---|
| | | 0 | 0.2 | 0.6 | 0.8 | 0.9 | 0.95 |
| Phase shift = 0; Amplitude = 0.3 | **r=0.01** | 0.01 (0) | 0.01 (0) | 0.01 (0) | 0.01 (0) | 0.01 (0) | 0.008 (-0.2) |
| | **r=0.05** | 0.048 (-0.04) | 0.048 (-0.04) | 0.048 (-0.04) | 0.049 (-0.02) | 0.047 (-0.06) | 0.042 (-0.16) |
| | **r=0.10** | 0.096 (-0.04) | 0.096 (-0.04) | 0.097 (-0.03) | 0.096 (-0.04) | 0.095 (-0.05) | 0.086 (-0.14) |
| | **r=0.20** | 0.192 (-0.04) | 0.193 (-0.035) | 0.192 (-0.04) | 0.193 (-0.035) | 0.191 (-0.045) | 0.171 (-0.145) |
| Phase shift = 0; Amplitude = 0.4 | **r=0.01** | 0.01 (0) | 0.009 (-0.1) | 0.01 (0) | 0.01 (0) | 0.01 (0) | 0.008 (-0.2) |
| | **r=0.05** | 0.05 (0) | 0.048 (-0.04) | 0.048 (-0.04) | 0.049 (-0.02) | 0.048 (-0.04) | 0.042 (-0.16) |
| | **r=0.10** | 0.099 (-0.01) | 0.095 (-0.05) | 0.096 (-0.04) | 0.097 (-0.03) | 0.098 (-0.02) | 0.082 (-0.18) |
| | **r=0.20** | 0.199 (-0.005) | 0.19 (-0.05) | 0.192 (-0.04) | 0.194 (-0.03) | 0.194 (-0.03) | 0.164 (-0.18) |
| Phase shift = 180; Amplitude = 0.3 | **r=0.01** | 0.009 (-0.1) | 0.009 (-0.1) | 0.009 (-0.1) | 0.009 (-0.1) | 0.009 (-0.1) | 0.008 (-0.2) |
| | **r=0.05** | 0.047 (-0.06) | 0.045 (-0.1) | 0.046 (-0.08) | 0.047 (-0.06) | 0.045 (-0.1) | 0.04 (-0.2) |
| | **r=0.10** | 0.094 (-0.06) | 0.091 (-0.09) | 0.092 (-0.08) | 0.094 (-0.06) | 0.089 (-0.11) | 0.081 (-0.19) |
| | **r=0.20** | 0.188 (-0.06) | 0.181 (-0.095) | 0.182 (-0.09) | 0.189 (-0.055) | 0.179 (-0.105) | 0.162 (-0.19) |
| Phase shift = 180; Amplitude = 0.4 | **r=0.01** | 0.009 (-0.1) | 0.009 (-0.1) | 0.009 (-0.1) | 0.009 (-0.1) | 0.009 (-0.1) | 0.008 (-0.2) |
| | **r=0.05** | 0.045 (-0.1) | 0.047 (-0.06) | 0.045 (-0.1) | 0.047 (-0.06) | 0.045 (-0.1) | 0.037 (-0.26) |
| | **r=0.10** | 0.089 (-0.11) | 0.093 (-0.07) | 0.09 (-0.1) | 0.093 (-0.07) | 0.09 (-0.1) | 0.075 (-0.25) |
| | **r=0.20** | 0.178 (-0.11) | 0.186 (-0.07) | 0.179 (-0.105) | 0.187 (-0.065) | 0.182 (-0.09) | 0.152 (-0.24) |

# References

[1] Klepac P, Caswell H. The stage-structured epidemic: linking disease and demography with a multi-state matrix approach model. Theoretical Ecology. 2011;4(3):301-19.

[2] Klepac P, Pomeroy LW, Bjørnstad ON, Kuiken T, Osterhaus ADME, Rijks JM. Stage-structured transmission of phocine distemper virus in the Dutch 2002 outbreak. Proceedings of the Royal Society B: Biological Sciences. 2009;276(1666):2469-76.

[3] Metcalf CJE, Lessler J, Klepac P, Morice A, Grenfell BT, Bjørnstad ON. Structured models of infectious disease: Inference with discrete data. Theoretical Population Biology. 2012;82(4):275-82.

[4] Winter AK, Martinez ME, Cutts FT, Moss WJ, Ferrari MJ, McKee A, et al. Benefits and challenges in using seroprevalence data to inform models for measles and rubella elimination. Journal of Infectious Diseases. 2018;218(3):355-64.

[5] Fine PEM. Herd immunity: History, theory, practice. Epidemiologic Reviews. 1993;15(2):265-302.

[6] Guerra FM, Bolotin S, Lim G, Heffernan J, Deeks SL, Li Y, et al. The basic reproduction number ($R_0$) of measles: a systematic review. The Lancet Infectious Diseases. 2017;17(12):e420-8.

[7] Finkenstädt BF, Grenfell BT. Time series modelling of childhood diseases: a dynamical systems approach. Journal of the Royal Statistical Society: Series C (Applied Statistics). 2000;49:187-205.

[8] Bjørnstad ON, Finkenstädt BF, Grenfell BT. Dynamics of measles epidemics: Estimating scaling of transmission rates using a time series SIR Model. Ecological Monographs. 2002;72(2):169-84.
