# Supplementary material for: Bias in the estimated reporting fraction due to vaccination in the time-series SIR model
Source: PLoS One. 2025 Aug 22;20(8):e0330568. doi: 10.1371/journal.pone.0330568 (PMC12373160; doi:10.1371/journal.pone.0330568)
Supplement: Attachment — Submitted filename: response_to_reviewers.docx [file pone.0330568.s011.docx]

Thank you for the opportunity to revise our manuscript PONE-D-24-22726 “Bias in the estimated reporting fraction due to vaccination in the time-series SIR model” for PLOS One. We have addressed each reviewer’s comments (in blue) and describe our changes below in our detail.

**Reviewer #1:**

This paper generates a time series of measles cases using an age-structured SIR transmission model with vaccination after birth and illustrates the bias with the standard TSIR fitting method.

The paper contains very interesting results and it can be accepted for the publication after some major revisions.
Comments:
1. In the introduction section, give more motivation and novelty of your study.

We have now emphasized the novelty of our study by adding that we “relax the assumption of vaccination at birth”. For emphasis of the motivation, we have added:

In countries where measles is uncommon, the recommended age for measles vaccination is later at 12 to 15 months, leaving a longer time gap still for the potential for double counting.

2. On page 2, add the biological meanings of each parameter of the model.

Thank you for pointing this out. We have added details in Section 2.1 to describe the model parameters.

3. Extend the model presented on page 2 to a nonlinear fractal-fractional system by using the Hattaf fractal-fractional derivative given in [1] in order to consider the memory effect which is an important characteristic of biological systems.

Thank you for the suggestion. We feel that extending the model to a nonlinear fractal-fractional system to consider the memory effect of biological systems would be beyond the scope of this work. Here our focus is on the estimation of reporting rate and total incidence using the TSIR framework.

4. Compare your results with others existing in the literature.

We have now added comparison of our results in context to existing literature through the discussion.

5. Include and comment in the manuscript the recent and related works:
[1] A new class of generalized fractal and fractal-fractional derivatives with non-singular kernels, Fractal and Fractional (2023).
[2] A new mixed fractional derivative with applications in computational biology, Computation (2024).
[3] A delayed SIR epidemic model with general incidence rate, Electronic Journal of qualitative theory of differential equations (2013).

Please see our comment to Point 3. We feel these references are beyond the scope of this work.

6. Improve your paper by adding the conclusion section and some prospects for your future research.

We have expanded the discussion on what identification of bias in the TSIR model in the presence of vaccination could mean for data collection.

7. There are some typos. The authors should carefully read the manuscript.

We have re-read and fixed some typos.

8. Check and unify the citation of references.

We have now fixed inconsistencies in the reference format.

**Reviewer #2:**

This is a paper investigating a methodological issue in applying the TSIR in the context of vaccination.

While I acknowledge that such methodological investigation is of interest, it would have been nice to have an example with real data so that it does not remain a toy. Did the question arise from considering a real situation?

Thank you for this suggestion. We agree that an example with real data would be a nice addition. For this, we use a dataset of rubella cases in Mexico and estimate the reporting ratio of all states in Mexico.

Section 2.4 in the Methods has been added to describe the data and what we did with the data. Results of this case study of rubella are now at the end of the Results section. The implications of this case study are in the Discussion.

I could not find the code/analysis to reproduce the results in the paper.
For such methodological works where all data are simulated, I don't see why the code would not be made available. It makes it easier to see the details of implementation.

Thank you for pointing this out. We forgot to add the link previously (now in Methods). The code is available on Github at https://github.com/TiffanyNL/tsir.

Specific points :

In paragraph 2.1, the equations are presented without definition of the notation. There could be a little more explanation here. It took me some time to understand that *presumably* there is no compartment to track the vaccinated immune, i.e. the compartments _v are those where vaccination failed rather than all vaccinated.

Thank you for pointing this out. In Section 2.1, we have added the differences between vaccinated and unvaccinated classes and also added the biological meaning of parameters and notations.

P3 - eq4. It would be nice to recall what linear relation there is between B and I.
is the cumulation on a birth cohort?

Thank you for the suggestion. We have added the linear regression relationship between B and I now (in Equation 6 and 7). Yes, the relationship is on the cumulative births and cumulative cases/infections.

par 2.3 "...fitting the the expanded…"
I don't feel that the fitting procedure is described in a way that it could be reproduced.
Is the 2 steps approach necessary? It seems that the model could be fit directly ?

Thank you for pointing out the typo. We have expanded the fitting procedure in the methods and made the code available.

par 3. "..under which violates the implicit assumption..." this is somewhat cryptic.

We have now clarified that we refer to the assumption of homogeneous mixing here.

Figure 1 : all symbols are triangles (upwards or downwards) - there is no square.
Is it OK to report absolute bias in the reporting rate? Does the problem show with all reporting rates? Here, the representation makes it as if there is no bias for small reporting rates? A Table may be more appropriate to present all this information.

Thank you for catching the lack of square symbol. We have simplified the figure and different shapes are no longer necessary to differentiate the various combinations of amplitudes/phase shifts/birth rates. The relative bias grows with vaccination coverage but not with reporting rate, and we have now added this to the results. We have also added a table of the reporting rate estimates and the relative bias in the supplementary.

Figure 2 : "unity" should be "identity". It is very difficult to see reporting fraction in this plot. Separate plots should be considered if this information must be showed. Also, if comparison is sought, a different regression line should be fit for each reporting rate.

Thank you for this. We have now replaced “unity” with “identity” throughout the manuscript. We were not comparing reporting rates but rather the regression lines for each vaccination coverage. We have emphasized this comparison accordingly:

The estimates of annual burden using different true reporting fractions do not differ significantly.

Overall, I think that the authors could simplify the figures, for example joining 1-3 and 2-4
to better show the gain, taking less scenarios and defering others to the supplementary.
Figure 5/6 could likewise be merged.

Thank you for this suggestion. We have combined figures 1 and 3 (estimated reporting fraction), figures 2 and 4 (estimated annual burden), and figures 5 and 6 (the estimated seasonality). These new figures now show 1 birth rate and 0 phase shift and for vaccination coverage 0, 60, 80, and 90%.

We have moved the analogous figures with a lower birth rate, 180-day phase shift, and the full range of vaccination coverage (0, 20, 60, 80, 90, 95%) to the supplementary.


Discussion
"The magnitude of this bias will increase when birth rates" true for absolute bias, but is it also true for relative bias?

Yes, it is true for both absolute and relative bias. In the same sentence, we now specify that both the absolute and relative bias increase when birth rates are high and when vaccination rates are high.
